# ‘Unmasking’ masked address data: A medoid geocoding solution

**DOI:** 10.1016/j.mex.2023.102090

**Published:** 2023-02-22

**Authors:** Edward Helderop, Jake R. Nelson, Tony H. Grubesic

**Affiliations:** a Center for Geospatial Sciences, School of Public Policy, University of California Riverside; b Department of Geosciences, Auburn University

**Keywords:** Open data, Privacy, Masked data, Geocoding, Spatial analysis, Medoid geocoding

## Abstract

In recent years, there has been a consistent push for more open data initiatives, particularly for datasets collected by public agencies or groups that receive public funding. However, there is a tension between the release of open data and the preservation of individual and household privacy, whose balance shifts due to increased data availability, the sophistication of analysis techniques, and the computational power available to users. As a result, data masking is a standard tool used to preserve privacy. This is a process in which the data publishers obfuscate some identifying features in the dataset while attempting to maintain as much accuracy and precision as possible. For spatial datasets, the geocoding of administratively-masked data has been a consistent problem. Here, we present a medoid-based technique that geocodes masked data while minimizing the spatial uncertainty associated with the masking approach. Unfortunately, many commercial geocoding software packages either fail to geocode administratively-masked data or provide false positives by assigning points to city or street centroids. We demonstrate the results of our medoid-based geocoding approach by comparing it to commercial geocoding software. The results suggest that a medoid geocoding approach is mechanically simple to deploy and maximizes the spatial accuracy of the resulting geocodes.

• Administratively-masked data are difficult to geocode
• A medoid geocoding method maximizes geocoding accuracy
• This method outperforms commercial geocoding software

## Graphical abstract

**Figure:**
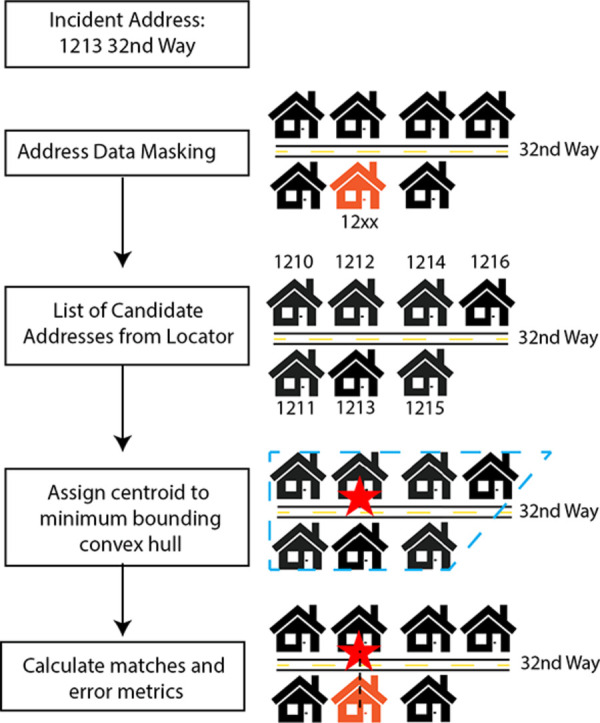

Specifications table

**Table:** 

Subject area:	Earth and Planetary Sciences
More specific subject area:	Geography
Name of your method:	Medoid geocoding
Name and reference of original method:	N/A (original method)
Resource availability:	Script publicly available on Github

## Method details

### Overview

Many federal, state, and local agencies mask datasets in one or more ways to provide anonymity to the individuals or households the dataset seeks to describe. This privacy-protecting approach is common whenever researchers obtain data at a highly granular level (e.g., individual or household). For example, it appears when dealing with healthcare data [1], business data (e.g., customer or employment records) [2], crime data [3], and census data [4], among many others. The range of masking approaches is as varied as the datasets that are being masked, with each approach ideally designed to maintain anonymity for the subjects while leaving some level of data analysis a possibility. This tension – between maintaining appropriate levels of individual privacy and allowing for the type of fine-grained analysis that the release of the data is supposed to enable – has been an actively debated issue for decades [5]. However, the recent explosion of publicly available spatial data has resulted in a wide array of masking techniques used, some more effective than others. The methods detailed in this paper focus on spatial, or geographic, masking. Specifically, geographic masking is intended to hide the specific locations of individual observations. Data attached to individual addresses, for example, is masked such that the specific address and household location are obfuscated in some way, maintaining household-level anonymity.

It is important to note that this masking may make household-level spatial analysis difficult (or impossible). However, one can aggregate these data into a larger geographic area, such as a neighborhood or a block group, for analysis. This aggregation is, in fact, the intended compromise behind releasing masked datasets [6]. Spatial analysis at an aggregated resolution (coarser than the original dataset) is possible, with the caveat that it requires the researcher to correctly assign each masked observation to the new geographic unit of analysis. However, depending on the specific masking technique used for a dataset, it may be challenging to accurately aggregate points to the respective areal unit of the observation. Furthermore, it is nearly impossible to determine the accuracy without knowing the actual (i.e., true) area membership of the set of observations. As a result, researchers routinely face a patchwork suite of masking techniques, with no clearly superior method to aggregate these data into larger areas for analysis. Additionally, many analysts rely on commercial geocoding software that always returns a geocoding result – even if that result is highly inaccurate. Alarmingly, the match scores (or other accuracy metrics) that these software packages employ are rarely suitable for administrative masking, often resulting in high-match score geocoding results that are in fact inaccurate.

There is a large corpus of literature that works to quantify the effectiveness of a given masking technique and to identify best-practice methods – those that minimize information loss while maintaining a given level of individual privacy or confidentiality [7]. Kounadi and Leitner [8], for example, developed techniques to quantify the spatial error associated with different geographic masking techniques, utilizing them to build a framework that someone interested in masking data could use to ensure spatial confidentiality. Meanwhile, as more datasets are made available and computational power is increasing, there is an arms race developing between masking techniques and the ability to unmask those data [9]. For instance, Lee et al. [10] found that it is possible to reverse engineer the likely point clusters that result in a given kernel density estimate for health data, violating the privacy requirements of the underlying dataset. Similarly, Seidl et al. [11] note that masking and privacy preservation become significantly more challenging with time-series data since trajectory data can be strongly identifying, and a simple geographic mask will no longer suffice to maintain individual confidentiality.

This paper aims to contribute to this body of work by highlighting several common masking techniques for spatially-explicit point data and introducing a medoid geocoding technique that outperforms commercial geocoding software when working with an administratively-masked dataset. Additionally, the medoid approach generates easily understood metrics that quantify how confident a user should be in the accuracy of the geocoding result.

## Method details

### Background

Spatially-explicit point data typically have their locations recorded in one of two ways – the most straightforward is that an event (i.e., a single dataset observation) will have a set of spatial coordinates attached to it (e.g., typically a latitude and longitude coordinate pair). The alternative is an affiliation between an observation and another piece of data that denotes location, most commonly an address. Moving between these two spatial representations is conceptually trivial and is known as address geocoding when determining the spatial coordinates of an address (and reverse geocoding when finding the address that matches a coordinate pair). In both cases, however, an address locator dataset is needed – this dataset connects addresses and spatial coordinates for an area. For many U.S. municipalities, these are usually parcel or address point datasets maintained by county assessor offices. These two types of spatial data representation lend themselves to two broad categories of location masking.

The first type of location masking focuses on point data with spatial coordinates and is known as *jittering* or *dithering*. Jittering involves the displacement of points using some process of translation or rotation such that the original (true) location of a point is changed. At its most basic, a jittering process will move each point in a dataset to a random location within the bounds of a predefined area of the point's original location. More sophisticated jittering techniques also exist. Rather than relying on a purely random process, these techniques apply rules limiting where one can move a point. For example, consider a process of jittering for observations of crime events in an urban coastal area – by definition, these crimes all occur on land. Applying a purely random jitter process might result in crimes occurring over water features. To alleviate this possibility, analysts can apply a spatial mask limiting the jitter results to only the land. This same process can occur for other bounding features or administrative units in the same vein. One usually employs this masking approach for continuous spatial phenomena – events occurring anywhere within a landscape. State-of-the-art jittering techniques are highly effective at preserving anonymity while maximizing the effectiveness of publicly-released datasets [12], [26]. However, these require some geographic or statistical expertise to implement effectively, which many organizations do not possess. As a result, it is common, particularly for government groups such as public utilities and law enforcement, to employ administrative masking (detailed below) instead. These issues are further compounded when datasets are released due to a Freedom of Information Act (FOIA) request. Small government agencies may be required to perform an ad-hoc masking procedure for a dataset that was not intended for public consumption without the expertise available to ensure they are employing an effective masking technique.

The second type of masking is more commonly applied to address data and involves straightforward address obfuscation, known as *administrative masking*. Many administrative or demographic datasets are masked with this approach, as they are often recorded at the household (address) level but require some anonymization to preserve privacy. In these cases, rather than first geocoding the data and then applying a standard jittering technique, masking efforts will instead anonymize portions of each observation's address. For example, a masking process might take ‘1300 Main St.’ and convert it to ‘13XX Main St.’ or ‘XX00 Main St’. This approach is inelegant when compared to spatial jittering. However, it is an easy process to implement, and it is also surprisingly common for many datasets with observations at the household level, likely because its implementation is easy and does not require any overtly spatial processes or familiarity with geospatial analytical techniques. However, unlike jittering, this technique can introduce significant difficulties for researchers interested in aggregating these observations to larger areal units for analysis.

For example, if a project is concerned with state or province-level analysis, the minor spatial error introduced by masking will become irrelevant. However, the spatial error introduced by the masking process can dramatically impact analysis when aggregating to a smaller unit such as the block, block group, neighborhood, or another similarly sized area. Specifically, the masking error may associate many observations with the incorrect spatial unit. Furthermore, it may be tempting to use non-masked portions of an address for aggregation – most commonly a ZIP code – but spatial analysis at the ZIP code level is fraught with problems [13], [14], [15]. Thus, more robust analysis is often possible at a similar scale to something like census block groups, but that requires careful treatment of the aggregation methods chosen. The remainder of this paper will introduce a medoid geocoding technique that minimizes spatial error when geocoding administratively-masked datasets.

### Medoid geocoding

Consider datasets like the crime data provided by the Cincinnati Police Data Initiative [[16] (CPD 2021] or water shutoff and restoration data from the Detroit Water and Sewerage Department [25]. The crime dataset is an incident dataset that contains data on reported crimes in and around the city of Cincinnati, Ohio. The use of these data and other datasets like it is pervasive in public policy [17], social science [18], criminology [19], [27], and other socio-economic planning sciences. The water utility data contain details on households that experienced water shutoffs and restorations for reasons like nonpayment. The respective agencies mask these datasets using different approaches. For example, the PDI removes the last two digits of the numeric street address for each crime event in Cincinnati. This masking approach generates entries such as ‘30XX Madison Rd.’ In this form, the given address reports each street accurately but does not disclose *where* the crime took place on the street. Meanwhile, the water utility data is masked by removing the first two address number digits, yielding: ‘XX00 Madison Rd.’

Address number masking introduces several problems. First, unlike jittered latitude and longitude coordinates that result in new (masked) coordinate pairs that are still plottable, masked addresses are not easily geocoded. Furthermore, the level of masking varies significantly between different observations, based on factors like street length, local parcel sizes, the number of properties on a given street, the number of digits in the street number, and the number of possible candidate addresses. In addition to solving the mechanical problem of geocoding these masked addresses, it is equally important to understand the level of uncertainty associated with each observation.

We begin with a master address point dataset (i.e., address locator) for the Cincinnati area, available from the Cincinnati Area Geographic Information System [24]. This dataset provides information on the true location of each address in the city and is the same dataset one would use for geocoding unmasked data [20], [28]. Using these data, one generates a list of ‘correct’ candidate addresses for each masked observation. Using the example of 30XX Madison St from the masked crime dataset, we use the master address database to identify all possible matches, including 3000 Madison St, 3010 Madison St, etc. These candidate addresses are generated using a string-matching process that uses the ZIP code, street name, and the intact portions of the street number to identify all possible matches. Of course, this candidate list size will vary depending on which addresses are actually used for a study area. With this Cincinnati dataset, for example, nearly 10% of the masked observations have only *one* candidate address, meaning that we can match that 10% of data points to the known correct candidate address as if no masking were in place.

**Fig. 1:**
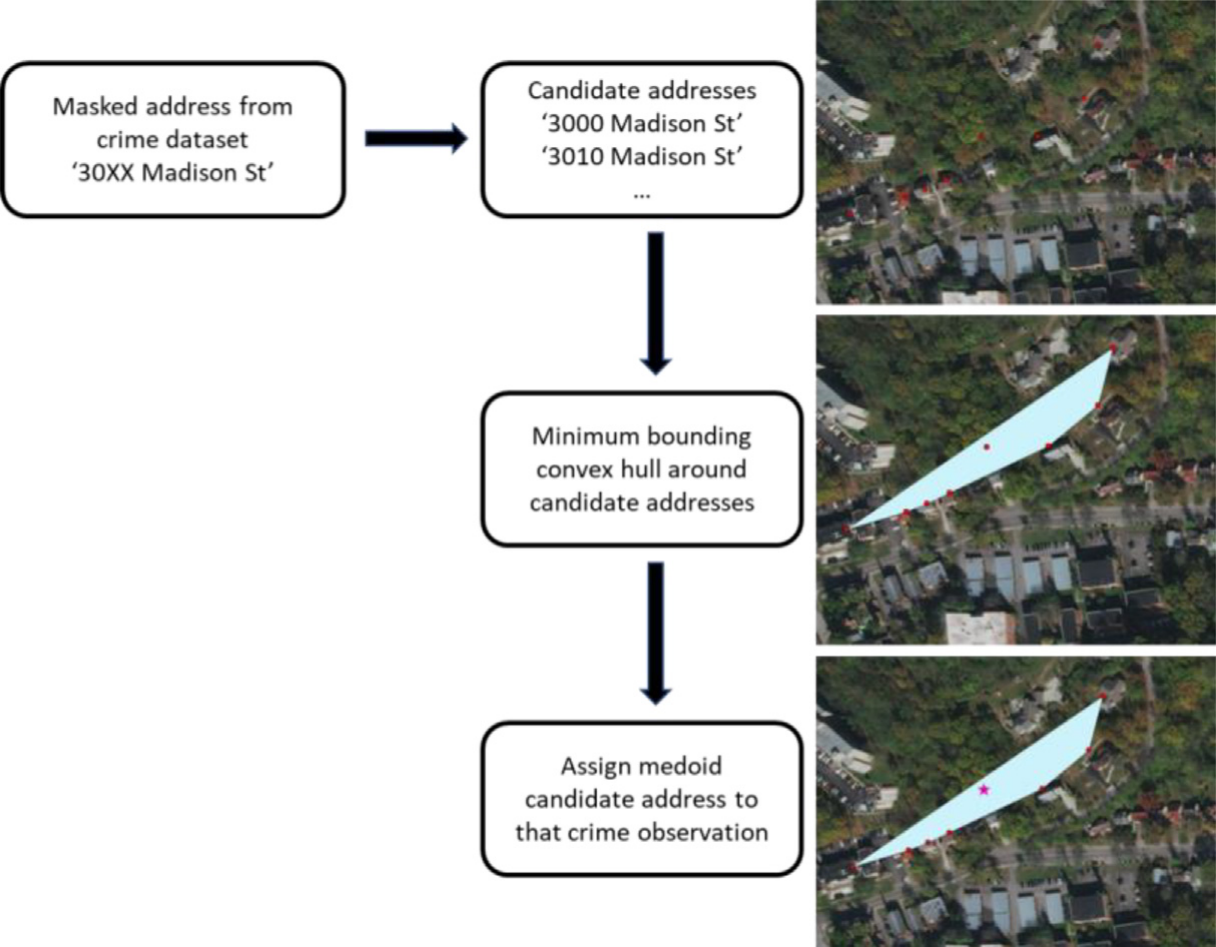
We pull all possible candidate addresses for each masked address in the crime dataset from a complete address point dataset for Cincinnati. Then, we calculate the convex hull for these points and assign the medoid candidate address as the 'correct' address for that crime observation.

For the remaining 90% of observations, there is more than one possible candidate address. Here we introduce the medoid-based geocoding technique to minimize the spatial error associated with this uncertainty. For spatial data, a medoid is the central-most data point that minimizes the summed distance from the medoid to all other data points. For each masked observation, a minimum bounding polygon is drawn around the list of candidate addresses. Within this polygon, one identifies the medoid and assigns this as the ‘correct’ address for the masked data point (Fig. 1). While no commercial software packages provide this tool, one can efficiently perform such tasks in R or Python. Traditionally, the most common method to select a given candidate address for a masked observation was to choose one randomly. However, depending on the spatial distribution of potential candidate addresses, a random selection has the potential to introduce large amounts of uncertainty. However, we note that for candidate lists of only two possible matches, no medoid exists, so we assign one to the masked observation at random in those cases. An example script has been made publicly available for users interested in adapting this technique to their own work.^1^

#### Confidence

Acknowledging that we structure the medoid-based approach to reduce the overall spatial error from geocoding masked addresses is vital. It is not a guarantee for finding the actual correct/true address for a masked observation. It is no more (or less) likely to be correct than one chosen at random. However, by identifying the medoid and constructing a minimum bounding polygon around the candidate addresses, this technique allows for a quantified measure of confidence in the geocoding result. This type of confidence measurement is essential for sensitivity analysis, in which a researcher may be more interested in excluding low-confidence geocodes rather than potentially introducing spatial error. It also allows researchers to examine potential outliers or anomalies in their analysis with greater detail and explain the results accordingly. For each medoid geocoded datapoint, the confidence is measured as *1/a* where *a* is the total area of the bounding polygon of the candidate addresses. A lower value means that the total area of the convex hull was larger, meaning there is less confidence that the medoid candidate is close to the true location of the masked data point.

### A comparison with commercial geocoding software

To test the medoid geocoding technique, we take a random sample of 10,000 addresses from a complete address shapefile of San Diego, California. Next, this dataset of 10,000 addresses was duplicated, with one copy being front-masked (i.e., the first two digits of each street number removed) and the other being back-masked (i.e., the final two digits of each street number removed). Both of these datasets were then medoid geocoded, using a custom Python script, and processed with three commonly-used geocoding software packages, including the classic commercial geocoding engine from Esri's Arcgis Pro geocoder [21], the U.S. Census Bureau's Census Geocoder [22], and OpenStreetMap's (OSM) Nominatim batch geocoding API [23].

Of the three, Esri's arguably performed the best, although still much worse than the medoid geocoding technique. We compare the results from Esri and the medoid technique in Table 1. Notably, all 10,000 observations in each of the two datasets were ‘successfully’ matched by Esri, with match scores ranging from just over 85 to 100. As can be seen below, the Esri match score is not a good geocoding accuracy metric for administratively-masked data. Meanwhile, the Census Geocoder performed the worst, with it having no flexibility to handle missing information. It returned a result of ‘No_Match’ for all 10,000 observations in each masked dataset. Nominatim performed slightly better, failing to match 64% of the observations across the two datasets. The remaining 36% resulted in median spatial error rates worse than the medoid geocoding technique but better than Esri. This result is likely because many of the particularly egregiously geocoded results from Esri were not matched by Nominatim. Because its median errors are calculated from a higher-confidence subsample of observations, we do not highlight these results.

**Table 1:** The percentage of correct matches and the median spatial error when geocoding a random sample of administratively masked address data using the medoid geocoding technique and Esri's ArcGIS Pro geocoding software. The medoid technique results in more correct matches, a lower spatial error, and a much higher percentage of correct block group assignment (the grey cells).

		Medoid geocoding		
Masking scheme	Example address	Correct matches	Median spatial error (m)	Correct Block Group assignment
*Front-masked*	“XX50 Main St.”	14.41%	680	78.02%
*Back-masked*	“10XX Main St.”	14.87%	712	81.11%

		Esri		
Masking scheme	Example address	Correct matches	Median spatial error (m)	Correct Block Group assignment
*Front-masked*	“XX50 Main St.”	2.60%	3631	62.30%
*Back-masked*	“10XX Main St.”	2.70%	6269	60.68%

**Fig. 2:**
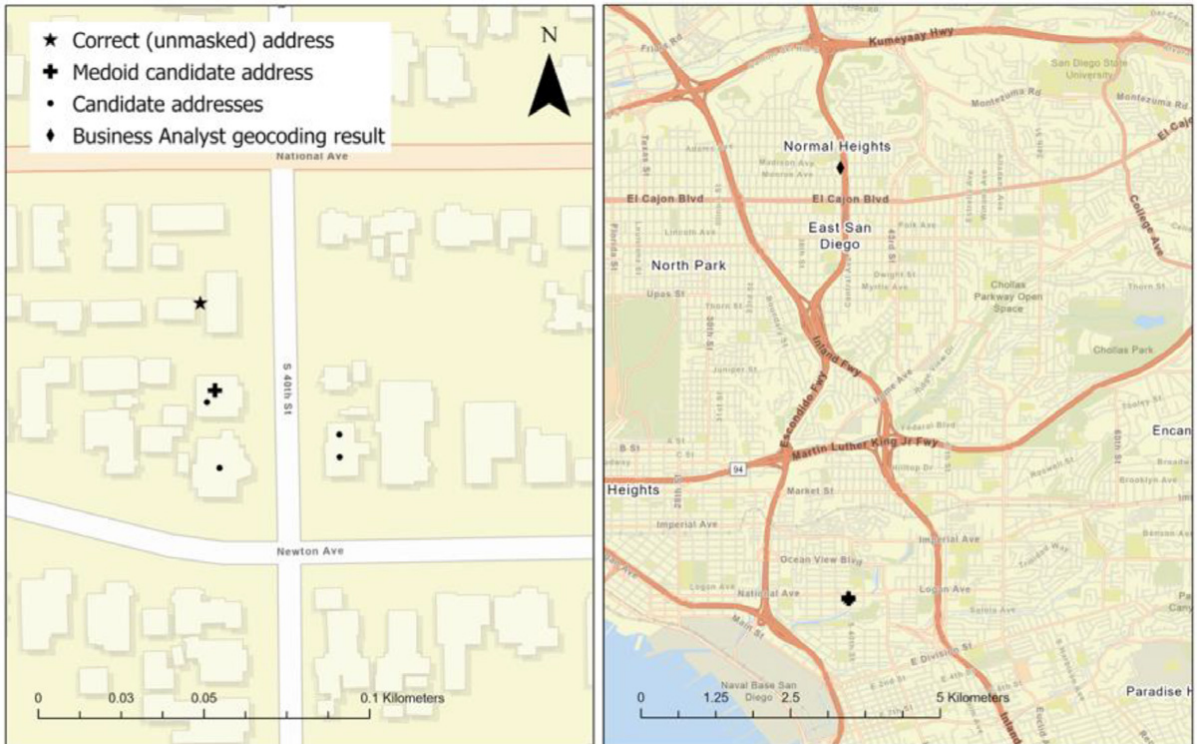
An example demonstrating how using commercial geocoding software can result in large spatial errors when working with masked data. Here, we see that ‘10XX S. 40^th^ St.’ has very few candidate addresses, so the medoid technique is robust. However, because Business Analyst cannot handle the ‘XX’ portion of the street number, it geocodes to an arbitrary point of 40^th^ St.

The results suggest that the medoid-based approach is more accurate than the classic commercial geocoding engine. Specifically, the number of correct matches is much higher. It is essential to acknowledge that this, at least in part, is due to the masked addresses having only one valid candidate address in the master database. However, it is also important to note that the median spatial error in the medoid-based approach is lower too. In addition, the percentage of masked geocoded addresses placed in their original block group is also higher. As previously mentioned, many socio-economic planning sciences aggregate event data (e.g., crimes) to a larger geographic unit of analysis. Errors in this aggregation can lead to over (or under) estimation of crime rates for these administrative units (e.g., blocks, block groups, census tracts). The results from this example illustrate how the medoid-based geocoding assigns a higher percentage of the masked point locations to the correct unit of analysis for both the front- and back-masked data. When exploring the matched addresses from the commercial geocoder, some of the error is likely because it has trouble interpreting street numbers that contain ‘XX’. For example, ‘XX9 Seaward Ave’ is matched to ‘9 Seaward Ave’, and ‘12XX Saint Cloud Way’ is matched to the street ‘Saint Cloud Way’ with no street number associated with the observation. In this case, the latitude and longitude coordinates correspond to the centroid of that street. This matching strategy can create enormous spatial errors for long streets (Fig. 2).

In Fig. 2, we highlight an example from our sample dataset. ‘10XX S 40^th^ St.’ is geocoded by the commercial software to an arbitrary point that falls along 40^th^ St, which is a long north-south arterial in East San Diego. It is interrupted several times by highways and has a large spatial footprint. The commercial geocoding result is over *seven kilometers* from the correct address (this geocoding result received a match score of 92). However, because the actual administrative blocks are geographically small, the medoid-based technique results in a high-accuracy geocode. Specifically, there are only six candidate addresses, and all of them are close to each other. In this case, the spatial error is only 87 meters. Furthermore, when examining the spatial distribution of addresses that were geocoded incorrectly by both Esri and the medoid approach, no obvious patterns present themselves. Observations with incorrect block group assignments are distributed evenly throughout the study area, for both types of masking. However, there is more overlap in the incorrectly geocoded observations between the two methods than would be expected if they were purely random. Were they purely random, one would expect around 800-900 observations to have been incorrectly geocoded by both approaches, depending on the masking type. Instead, there are nearly 1,300 observations that received the incorrect block group assignment by both geocoders. While a full exploration of this deviation is outside the scope of this paper, it likely indicates that these addresses are found along the edges of block groups.

Insidiously, however, the commercial software package fails to warn users about the small set of interpretable street numbers. Ideally, analysts relying on this type of geocoding package would perform appropriate due diligence and examine the results for sources of error. However, naïve users may not do so, never realizing the extent of the error in their geocoded results (a user who only relied on Esri match scores would have erroneously high confidence in their geocoding results here). To Nominatim's credit, it is worth noting that its match rate of 36% may actually aid researchers here, since it appears to use a match score/accuracy metric that is better able to measure when its geocoder cannot handle administratively-masked data. Esri, meanwhile, reports that it successfully matched all 10,000 observations in both datasets with match scores equal to or greater than 85, even in cases where there should be little confidence in the resulting geocode. It is easy to ignore this source of error when aggregating to a larger area, like a census block group. Most analysts view this as an appropriate way to minimize the spatial error associated with masked point data, allowing for easy integration of demographic information. However, in our example, over one-third of the commercially geocoded observations have been assigned to the wrong block group, compared to approximately one-fifth with medoid-based geocoding. Again, in an actual study lacking a reference dataset with cadastral information, for example, it would be impossible to know the extent of this error. Practitioners can perform similar tests using toy datasets in their study areas or rely on the confidence metric as a proxy for the maximum possible spatial error associated with the medoid geocoded observations to begin quantifying their spatial error.

## Conclusion

This paper introduced a novel medoid geocoding technique that works to geocode administratively-masked address data while minimizing the spatial uncertainty associated with the masking process. As illustrated previously, commercial geocoding software often fails to geocode masked address data or supplies users with false positives by simply geocoding to city or street centroids when the street number is obfuscated. For each masked datapoint, we highlighted a spatial confidence metric such that users can exclude low-confidence geocode results from their analysis if they choose. Finally, we note that there are ethical issues at play regarding de-anonymizing data to any degree. As we noted above, portions of many masked datasets can often be entirely unmasked. Therefore, it is incumbent upon researchers to preserve individual- or household-level anonymity in any deliverable (e.g., publication) or re-releases of datasets. To that end, we recommend that researchers aggregate the geocoded data to a larger area (such as a block or block group) or, if using point-level analysis, to ensure that the de-anonymized data is not released publicly.

## CRediT author statement

**Edward Helderop:** Conceptualization, methodology, software, formal analysis, writing - original draft, writing - review and editing

**Jake R. Nelson:** Conceptualization, resources, data curation, writing - original draft

**Tony H. Grubesic:** Validation, resources, supervision, writing - original draft

## Declaration of Competing Interest

The authors declare that they have no known competing financial interests or personal relationships that could have appeared to influence the work reported in this paper.

## Data Availability

The authors do not have permission to share data.

## Footnotes

1 https://github.com/Ehelderop/medoidGC.
